# Nuptial psychosis and Tunisian culture: A case report

**DOI:** 10.1192/j.eurpsy.2021.1804

**Published:** 2021-08-13

**Authors:** E. Mhiri, F. Charfeddine, N. Messedi, A. Chamseddine, W. Bouattour

**Affiliations:** 1 Psychiatry (b), Hedi Chaker University hospital, sfax, Tunisia; 2 Psychiatry, Hedi Chaker University hospital, sfax, Tunisia

**Keywords:** nuptial, psychosis, bride, culture

## Abstract

**Introduction:**

In Tunisia, traditions of marriage are still preserved by certain regions of the country : the taboo of sexuality and the requirement of the preservation of virginity until marriage mark the Tunisian mentality till this day.

**Objectives:**

To discuss the impact of the Tunisian culture related to marriage on the precipitation of psychotic disorders in the bride.

**Methods:**

A case report and a review of litterature via PubMed using the terms : « nuptial, psychosis, bride».

**Results:**

A 31-year-old woman With no personal desease, developped a mutism, refusal of food and heteroagressiveness since the day after the wedding. The wedding party went well and it was consumed on the first night without any real incidents, yet, Ms. H was very anxious about the loss of her virginity and especially because of the low bleeding she had. The day after the wedding day, the bride was especially worried because of the presence of her family waiting in front of the bedroom to see the the blood-stained sheet : proof of the virginity of their daughter and the virility of the husband. In fact, incertain of the reaction of the family, the patient left her house early without informing her husband and was found by the police. Later, she developped an incoherent speech, audio-visual hallucinations and delusions against those around her.

**Conclusions:**

In Tunisia, despite the progress made in terms of equality betwen men and women, women’s sexuality still suffer from certain taboos. Sexual education needs to be improved among young people to avoid subsequent sexual problems.

**Disclosure:**

No significant relationships.

